# Study of the factors influencing the use of MyData platform based on personal health record data sharing system

**DOI:** 10.1186/s12911-022-01929-z

**Published:** 2022-07-15

**Authors:** Wona Choi, Se-Hyun Chang, Yoon-Sik Yang, Surin Jung, Seo-Joon Lee, Ji-Won Chun, Dai-Jin Kim, Woonjeong Lee, In Young Choi

**Affiliations:** 1 Department of Medical Informatics, The Catholic University of Korea College of Medicine, 222 Banpo-daero, Seocho-gu, Seoul, 06591 Republic of Korea; 2 Department of Psychiatry, Seoul St. Mary’s Hospital, The Catholic University of Korea College of Medicine, Seoul, Republic of Korea; 3 Department of Emergency Medicine, Incheon St. Mary’s Hospital, The Catholic University of Korea College of Medicine, Incheon, Republic of Korea

**Keywords:** Personal health record, Information dissemination, eHealth, Health information system, Information services

## Abstract

**Background:**

The application of telemedicine and electronic health (eHealth) technology has grown in importance during the COVID-19 pandemic, and a new approach in personal data management and processing MyData, has emerged. Data portability and informational self-determination are fundamental concepts of MyData. This study analysed the factors that influence acceptance of the MyData platform, which, reflects the right to self-determine personal data.

**Methods:**

The study involved participants having experience using the MyData platform, and the key factors of the unified theory of acceptance and use of technology were used in the research model (performance expectancy, effort expectancy, social influence, facilitation condition and behavioural intention to use). The questionnaire comprided 27 items, and system usage log data were used to confirm that behavioural intention to use affected actual use behaviour through structural equation modeling.

**Results:**

In total, 1153 participants completed the survey. The goodness of fit in the structural equation model indices indicates that the data fit the research model well. Performance expectancy, social influence, and facilitating conditions had direct effects on behavioural intention to use. We used system usage log data to confirm that behavioural intention to use positively affected actual use behaviour. The impact of the main factors in the unified theory of acceptance and use of technology was not moderated by age or gender, except for performance expectancy.

**Conclusions:**

This study is the first to examine the factors influencing the use of the MyData platform based on the personal health record data sharing system in Korea. In addition, the study confirmed the use behaviour of the MyData platform utilising the system’s actual usage log for each function and analysing the effect of the intention of use on actual use. Our study serves as a significant foundation for the acceptance of data portability and sharing concepts. It also lays the foundation for expanding the data economy and ecosystem in the pandemic era.

**Supplementary Information:**

The online version contains supplementary material available at 10.1186/s12911-022-01929-z.

## Introduction

The application of telemedicine and eHealth technology in clinical services is gaining importance in response to the COVID-19 pandemic [1]. New digital and mobile technologies point to a future in which consumers will be more involved in managing their health and personalised solutions [2]. Systems, such as personal health records (PHRs), have been developed to allow individuals to better manage their own health. However, despite these expectations, there is insufficient evidence that PHRs increase patient engagement or improves clinical outcomes [3, 4].

A digital infrastructure that collects, processes, distributes and utilises personal health data has been suggested to enable new combinations of digital and physical components that produce innovative eHealth services [5]. MyData is a new approach in personal data management and processing [6]. Data portability, defined as users’ ability to transfer their personal data to different online platforms, is the fundamental concept of MyData [7]. Data portability increases the informational self-determination of data, by providing the data subject with control over personal data [8].

On 9 January 2020, the National Assembly of the Republic of Korea announced, that it had passed proposed amendments (collectively, ‘the Data 3 Act’) to the Personal Information Protection Act 2011 ('PIPA'), the Act on Promotion of Information and Communications Network Utilization and Information Protection 2001 ('ICNA'), and the Credit Information Use and Protection Act 2008 ('the Credit Act') [9]. The right to data portability indicates the legal possibility of data transfer, opening the doors to providers to attract users with even more personalised services [8]. Based on the Data 3 Act, a country-led MyData project was undertaken in South Korea that led ti the development of a MyData platform called ‘HiMD’ [10].

Information technology using the internet and mobile devices provides numerous advantages in the healthcare sector [11]. Understanding the factors that influence technology acceptance is essential for successful adoption [12]. With regard to information systems and communication technology, research models have been developed to understand these factors and predict technology acceptance, in particular the information system success model, the technology acceptance model (TAM), and the unified theory of acceptance and use of technology (UTAUT) [13–16]. Technology acceptance is a relatively mature research area that has received considerable attention in various areas, and has been the subject of extensive research in the healthcare fields [17–22]. However, many studies of the technology acceptance in the healthcare field have been restricted to investigations before technology experience of use [5, 23]. Very few studies have investigated users’ acceptance of the MyData platform, in which data portability is the fundamental concept and basis of the personal health record data sharing system. According to previous studies, increasing the use of patient health data requires an interconnected architecture and a patient-centric design [24, 25]. This is also important for understanding the consumer perspective of the new healthcare system. A better understanding of health consumers’ intentions and behaviours would help to develop the system and implement effective and efficient strategies [26].

Therefore, this study aimed to analyse the factors influencing acceptance of the MyData platform, which reflects the right to self-determine personal data. In this study, we used the key factors of UTAUT in our research model and applied structural equation modeling (SEM) to analyse the relationships between the variables of the framework in order to determine the factors influencing the intention to use and actual use of the platform.

## Related works

The suggested UTAUT model is known to have greater explanatory power than the TAM, which, on average, exhibits 40–50% explanatory power regarding the end user's behaviours or behavioural intention to use. Based on this, UTAUT has been empirically validated and used as a basis for research related to technology acceptance in various fields. In addition, studies that have applied this model have proven its flexibility and scalability. A similar theoretical framework was employed to develop the hypotheses [16, 27, 28].

### Related studies

Andrew et al. studied the perceptions and beliefs of a personally controlled electronic health record (PCEHR) in Australia, via an online survey applying technology acceptance models. Perceived value and perceived risk were the two most important variables explaining attitudes towordthe PCEHR, and the participants did not appear to consider it particularly useful at that time [29].

Koivumäki’s study (2017) aimed to investigate the factors influencing consumers’ intentions to use a MyData-based preventive eHealth service, using a new adoption model combining UTAUT2, threat appraisals, self-efficacy, and perceived barriers. Most of the hypotheses related tothe explanatory UTAUT2 constructs were rejected, and the other three health motivation constructs(threat appraisals, self-efficacy and perceived barriers) significantly affected behavioural intention. This study focuses on increasing the understanding of the factors influencing consumers' eHealth technology acceptance [5].

Although many studies of issues in the healthcare system, including PHR acceptance, have been conducted using the UTAUT model, they have largely been limited to 'intention to use'; studies confirming 'actual use behaviour' were very rare. We conducted a study of a system with actual users, which can increase the reliability of the acceptance factors for the system [30, 31].

Actual usage behaviour was measured as the duration of use via system logs in the original study on UTAUT. Other studies that investigated usage behaviour used questionnaires as measurement tools [32, 33]. Previous studies of UTAUT in relation to the healthcare system have tended to focus on self-reporting rather than on the system log data of patients. This study handled the users’ subjective data using survey data, and objective data using system log data.

### The MyData platform based personal health record data sharing system

Since the establishment of a data ecosystem that actively utilises personal information, related policy discussions have continued regarding data sharing and utilisation [34, 35]. In addition, country-led projects reflecting data portability have been ongoing in the UK, Sweden, Australia, and South Korea [10, 29, 36, 37].

The MyData Platform called HiMD manages the sharing of data from PHRs and supports customised health care services. The system’s primary functions include consent, data checking, data downloading, data sharing, and data transfer. Consent settings consisted of service terms for companies, data sharing with third parties, data transfer, and data use. Users can make decisions regarding their own data at every step of the way and check the flow of data within the HiMD [10]. The four companies participating in the MyData platform provide personalised healthcare services using shared data genome analysis services for cancer, chronic disease management services, customised meal kit services, and mental health management services. Figure 2 shows the screen of the HiMD application: (a) services provided by the companies, (b) management of the service consent and (c) data usage receipt.

## Theoretical framework and hypothesis development

### Unified theory of acceptance and use of technology

**Fig. 1:**
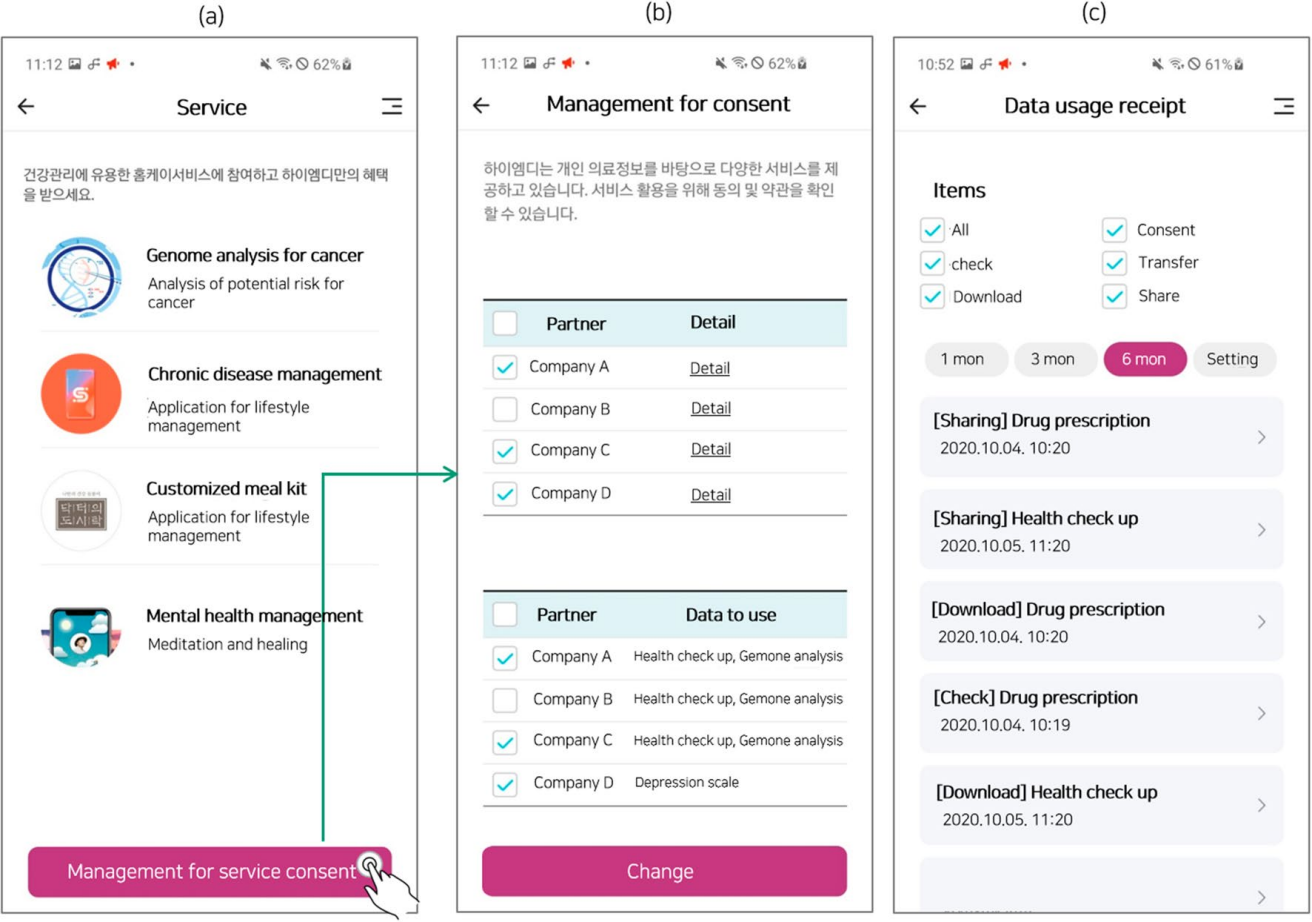
HiMD: MyData platform based on the PHR data sharing system [10]

**Fig. 2:**
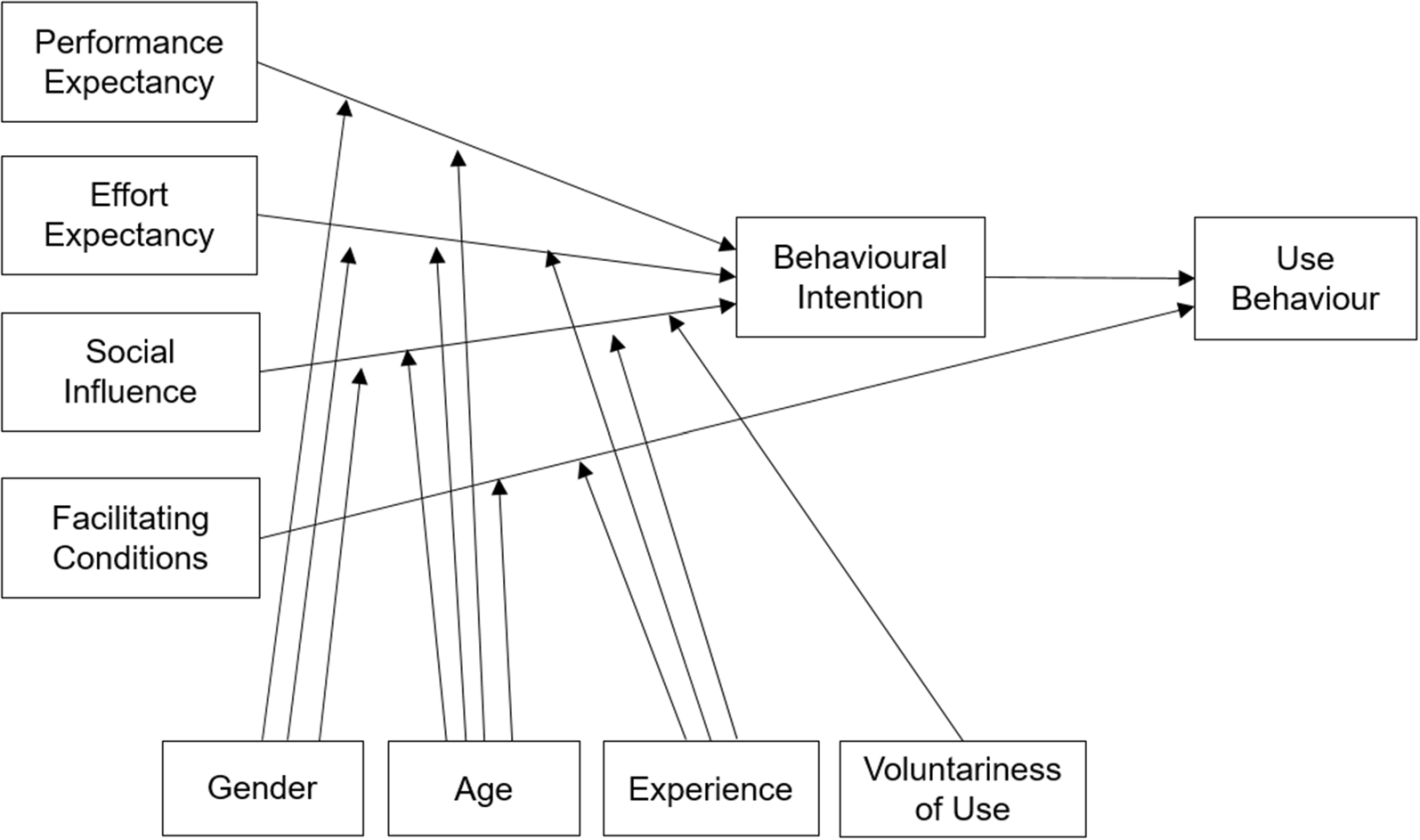
The unified theory of acceptance and use of technology model [15]

TAM, initially developed by Davis [14], is used to analyse technology acceptance and conceptualise expressions of human behaviour. Subsequently, to further understand technology adoption, Venkatesh et al. [15] combined and elaborated on eight models to develop the UTAUT (Fig. 1), which has better explanatory power than previous models. The UTAUT model has four fundamental constructs (performance expectancy, effort expectancy, social influence, and facilitating conditions) that influence behavioural intention to use technology and/or technology use, with four moderating variables (gender, age, experience, and voluntariness of use). The UTAUT model posits that performance expectancy, effort expectancy, and social influence affect behavioural intention to use technology, while behavioural intention and facilitating conditions determine technology use. Consumer acceptance of a technology is determined by the intention to use, which leads to the actual use of the technology [16] (Fig. 2).

### Research model

**Fig. 3:**
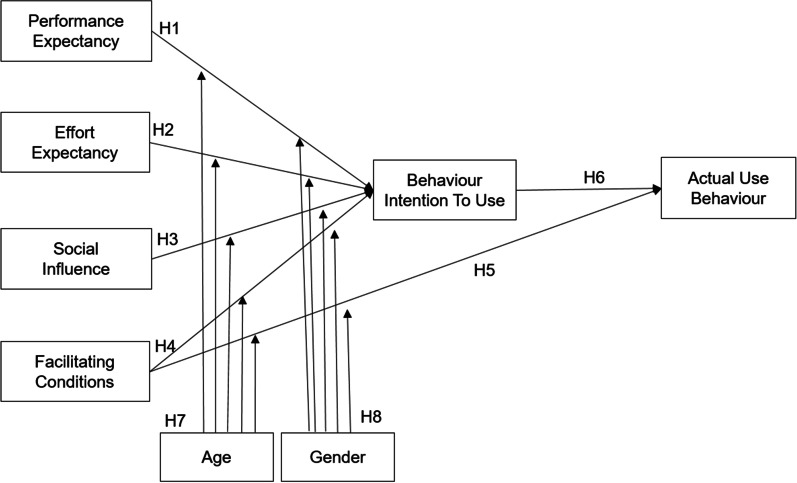
Proposed research model

This study aimed to investigate a preventive eHealth service called ‘HiMD’, a PHR data sharing system based on MyData. The UTAUT is used as a theoretical foundation for implementing the research model in this study to choose a model that includes the factors that influence users' intention and adoption of the MyData platform in Korea. We then,extracted data on the actual use behavior from an actual system log. Age and gender were also assumed to moderate the effects of each factor. Figure 3 illustrates the conceptual research model used in this study.

### Research hypothesis

The original UTAUT model suggests that performance expectancy, effort expectancy, and social influence positively influence behavioural intention to use, and that facilitating conditions positively influence use behaviour [15]. UTAUT2 has been shown to be more explanatory in predicting behavioural intentions [16, 38, 39]. In addition, to study the acceptance of technology, researchers have expanded the research model by combining various factors, with the UTAUT model [40, 41]. However, the additional factors in UTAUT (hedonic motivation, price value and habit) are not applicable to the HiMD platform; therefore, we decided that the factors of UTAUT are appropriate for this system and focused on the original UTAUT model.

#### Performance expectancy (PE)

Performance expectancy(PE) refers to the system’s usefulness when used for a specific purpose. In other words, PE is defined as the degree to which users believe that using HiMD will help them improve their health [15]. PE is the strongest predictor of behavioural intention with respect to adopting new technology, according to the literature [42]. Therefore, we propose the following hypothesis:

##### H1

Performance expectancy in HiMD has a positive effect on behavioural intention to use.

#### Effort expectancy (EE)

Effort expectancy(EE) refers to “the extent of ease connected with the use of a system”. This is the degree of ease associated with the use of HiMD in this study [15]. Users have been reported to be more likely to apply a new system if they find its adoption easy [43]. Thus, we propose the following hypothesis:

##### H2

Effort expectancy in HiMD has a positive effect on behavioural intention to use.

#### Social influence (SI)

Social influence (SI) is defined as the degree to which users feel that important relatives or colleagues believe that HiMD should be used for enhanced health management [15]. Different social groups, such as friends, family members, relatives, neighbours, acquaintances, and others who use technology, have a significant influence on users' perceptions and attitudes. Therefore, the following hypothesis on SI was proposed.

##### H3

Social influence in HiMD has a positive effect on behavioural intention to use.

#### Facilitating conditions (FC)

Facilitating conditions(FC) are the degree to which users believe that an organizational and technical infrastructure exists to support the use of HiMD [15]. This means that infrastructure and organizational support are necessary to use the information system. FCs have been shown to exert a considerable impact on the intention to use and the use behaviour of the health information system [15, 44].

##### H4

Facilitating conditions in HiMD have a positive effect on behavioural intention to use.

##### H5

Facilitating conditions in HiMD has a positive effect on actual use behaviour.

#### Actual use behaviour

Owing to the difficulty of investigating actual use actions, many studies have hypothesised that facilitating conditions influence behavioural intention to use [45, 46]. However, this study investigated the use actions by examining the participants' actual usage logs and hypothesised that facilitating conditions positively impacted both behavioural intention to use and actual use behaviour of the system. We referred to similar studies that have extended such concepts [28].

##### H6

Behavioural intention to use in HiMD has a positive effect on actual use behaviour.

#### Age and gender

Age and gender are generally perceived as significant factors in attitudes towards information technology [47]. Previous studies have also treated age and gender as moderating variables in technology acceptance [15, 30, 48]. We confirmed age and gender as moderating factors for intention to use.

##### H7

Age moderates the effects of performance expectancy, effort expectancy, social influence, and facilitating conditions on behavioural intention to use in HiMD.

##### H8

Gender moderates the effects of performance expectancy, effort expectancy, social influence, and facilitating conditions on behavioural intention to use in HiMD.

## Methods

### Development of measures

The questionnaire consists of 27 items. Responses to the 21 items measuring the main variables (performance expectancy, effort expectancy, social influence, facilitation conditions, intention to use)are scored a 5-point Likert scale ranging from *‘strongly disagree’* to *‘strongly agree’*, and 6 items pertain to participants’ general characteristics (age, gender, education, income, experience) [14, 15, 49–51]. The factor items are presented in Additional file 1: Tables S1 and S2.

Actual use behaviour was measured using the system usage log record, which consists of a specific function for HiMD. The functions are consent, data check, data download, data sharing, and the log for each function, which can be checked in the system and user applications (see Additional file 1: Table S3) [10]. Scores were added according to the degree of use of system functions. For example, three points were given if the user used three of four categories (consent, data check, data download, and data sharing).

### Data collection

Data were collected using a quantitative, mobile-based questionnaire survey and system usage logs. The HiMD platform was applied to people visiting the health examination centre of university hospitals and we conducted a survey of users with experience using the HiMD system for more than four weeks. The survey link was sent via an application push alert which connected participants to a mobile web page. The mobile web page included the survey for HiMD, with a cover letter explaining the research phenomenon and context, the purpose of the survey, the use of data, and the consent of the study. To prevent duplicate responses, each user could participate in the survey only once. As a result, the questionnaire received responses from 1153 users’. The link for the survey was open for three months (from 2 November 2020 to 1 February 2021). We analysed the usage log containing consent, data check, data download, and data sharing in the HiMD to check the actual use behaviour. The event log and information from the HiMD system were automatically stored in a database to enables users to check their logs in the HiMD application.

The study was conducted in accordance with the guidelines of the Declaration of Helsinki and approved by the Institutional Review Board of the Catholic Medical Center (protocol code XC20QIDI0145K and 13th October 2020). Informed consent was obtained from all the subjects involved in the study.

### Analysis

In general, SEM investigates the relationships between latent variables, measured by several items, and allows questions that involve multiple regression analyses of factors to be answered [52]. We employed SEM using the R (v4.1.0) software to evaluate the proposed research model. When we tested the moderating factors (age and gender), we estimated the significances of differences using a chi-square difference test through two models (one controlled and the other uncontrolled for each moderator).

Before conducting SEM analysis, we confirmed the reliability and validity of the constructs. Varimax rotation of factor loadings was used to identify the variables associated with each factor, and a cut-off value of 0.5 was used to extract the variables associated with each factor.

It is widely accepted that the ability to accept information technology differs depending on users' characteristics [45, 46]. We analysed the moderating effects of age and gender on the relationships between the constructs as moderators. We then performed a multi-group path analysis to verify the moderating effects of gender that supported the moderator, except for age.

## Results

### Participant characteristic

The study involved 1153 participants who had experience using the HiMD system for over four weeks. The general characteristics of the participants are summarised in Table 1. The participants comprised 234 men (20.3%) and 919 women (79.7%) More than 60% of the sample was represented by those in their 20 s and 30 s (67.3%), followed by those in their 40 s (20.0%), and 50 s (12.6%). Of the participants, 95.4% were college graduates or had higher educational qualifications.

**Table 1:** Characteristic of the participants

Characteristics	n	%	Cumulative %
*Gender*			
Male	234	20.3	20.3
Female	919	79.7	100.0
*Age (year)*			
20–29	343	29.7	29.7
30–39	434	37.6	67.3
40–49	231	20.0	87.3
≥ 50	145	12.6	100.0
*Education*			
Middle school	2	0.2	0.2
High school	51	4.4	4.4
College	858	74.4	79.0
Graduate school	242	21	100.0

### Reliability and validity

The results of the exploratory factor analysis (EFA) and parallel analysis scree plot indicated the appropriateness of the factor analysis (see Additional file 1: Table S4 and Fig. S1).

Additional file 1: Table S5 shows the results of the reliability and confirmatory factor analysis (CFA). Cronbach’s alpha values greater than 0.60 are generally considered acceptable and values greater than 0.90 highly reliable. Cronbach’s alpha, which indicates the reliability of the main variables, was considered acceptable within the recommended range (> 0.70). To check convergent validity, we analysed the values, including the standardised factor loading, significance, average variance extracted (AVE), and construct reliability (CR). When the CR value is ≥ 0.7 and the AVE value is 0.5, the measured variables are considered valid. The CR value for all constructs was higher than 0.7, thereby confirming the reliability of the model [53]. Therefore, all the factors were deemed fit for analysis. Several fit indices were used to evaluate CFA model fit. The fit indices of the research model were calculated as follows: χ^2^ = 897.244 (*df* = 179, *p* < 0.001), CFI = 0.976, TLI = 0.971, RMSEA = 0.059 and SRMR = 0.024. All indices of the measurement model fit satisfied the recommended thresholds, thereby indicating that the model fit the data [54].

Discriminant validity was shown to analyse the correlations and the square root of AVEs (diagonal) of constructs and the correlation matrix plot (Additional file 1: Table S6 and Fig. S2).

A relationship between PE and SI is observed but it is lower than that of the diagonal components. The correlation coefficient between the items of the factors was checked, but the correlation coefficient of each item between PE and SI is under 0.8 (max = 0.71).

### Results from the SEM

Table 2 presents the results of the path analyses with standardised coefficients. The SEM results indicate that the model acceptably fit the data (χ^2^ = 935.57, *df* = 198, *p* < 0.001, CFI = 0.975, TLI = 0.971, RMSEA = 0.057, SRMR = 0.025).

**Table 2:** The result of the tested hypothesis

	Path			Coefficient	SE	*p*-value	Result
H1	PE	→	BI	0.271	0.039	< 0.001	Supported
H2	EE	→	BI	0.012	0.025	0.623	Not supported
H3	SI	→	BI	0.493	0.04	< 0.001	Supported
H4	FC	→	BI	0.221	0.033	< 0.001	Supported
H5	FC	→	AUB	− 0.052	0.043	0.223	Not supported
H6	BI	→	AUB	0.079	0.039	0.043	Supported

Goodness of fit: χ^2^ = 935.57, *df* = 198, *p* < 0.001, CFI = 0.975, TLI = 0.971, RMSEA = 0.057, SRMR = 0.025

PE: Performance expectancy, EE: effort expectancy, SI: social influence, FC: facilitating conditions, BI: behavioural intention, AUB: actual use behaviour

**Fig. 4:**
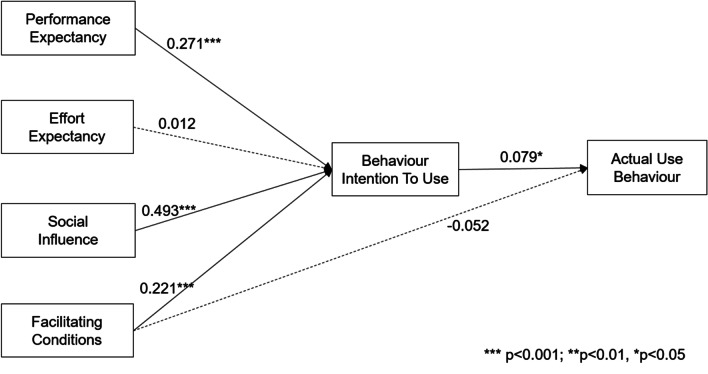
Path coefficients of the tested hypothesis

The results showed that performance expectancy (PE; β = 0.271, *p* < 0.001), social influence (SI; β = 0.493, *p* < 0.001), and facilitating conditions (FC; β = 0.221, *p* < 0.001) significantly positively affected behavioural intention to use (BI). Meanwhile, the effect of effort expectancy (EE) on behavioural intention to use (BI; β = 0.012, *p* > 0.05) and the effect of facilitating conditions (FC) on actual use behaviour (AUB; β =  − 0.052, *p* > 0.05) were not confirmed. Behavioural intention to use positively affected actual use behaviour (AUB) for HiMD (β = 0.079, *p* < 0.05). Therefore, the results supported H1, H3, H4, and H7, and were partially consistent with the original UTAUT model (Table2). The path analysis of the hypotheses is presented in Fig. 4.

**Fig. 5:**
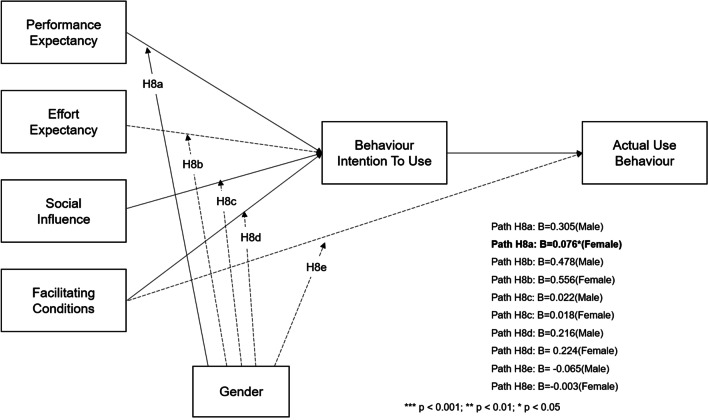
The result of moderating effect test for gender

Table 3 presents the results of the measurement invariance test for age and gender as moderators. Each row shows the test results for each hypothesis (Table 4). In the male group, effort expectancy had significant positive effects on behavioural intention to use, and H8 was partially supported. The moderating effects of the UTAUT are shown in Fig. 5.

**Table 3:** Measurement invariance test for gender and age groups

	Model	χ^2^ (*df*)	CFI	TLI	RMSEA	∆χ^2^ (∆df) Pr (> χ^2^)	Result
H7 (Age)	Non-restricted	1430.955 (522)	0.969	0.965	0.055	60.656 (33)	Not supported
	Full-metric invariance	1491.611 (555)	0.968	0.966	0.054	*p* = 0.00234	
H8 (Gender)	Non-restricted	1437.708 (522)	0.9690	0.9650	0.0550	34.909 (33)	Supported
	Full-metric invariance	1472.617 (555)	0.9690	0.9670	0.0540	*p* = 0.377

**Table 4:** Moderating effect test for gender

	Path			Male	Female	∆χ^2^ (∆df)	*p*-value	Result
H8a	PE	→	BI	0.305 (***)	0.076	4.914 (1)	0.027	Supported
H8b	EE	→	BI	0.478 (***)	0.556 (***)	0.638 (1)	0.425	Not supported
H8c	SI	→	BI	0.022	0.018	0.003 (1)	0.960	Not supported
H8d	FC	→	BI	0.216 (***)	0.224 (***)	0.007 (1)	0.934	Not supported
H8e	FC	→	AUB	− 0.065	− 0.003	0.319 (1)	0.572	Not supported

Fit indices of gender group model: χ^2^ = 1472.614, *df* = 555 *p* < 0.001, CFI = 0.969, TLI = 0.967, RMSEA = 0.054, SRMR = 0.04; * *p* < 0.1, ** *p* < 0.05, *** *p* < 0.01

PE: Performance expectancy, EE: effort expectancy, SI: social influence, FC: facilitating conditions, BI: behavioural intention, AUB: actual use behaviour

## Discussion and conclusions

### Principal results

This study aimed to analyse the factors influencing the acceptance of the MyData platform ‘HiMD’ leading to actual use behaviour.

This study set the main variables of performance expectancy, effort expectancy, social influence, and facilitating conditions as factors affecting behavioural intention to use and actual use behaviour based on the UTAUT model. We surveyed 1153 participants who used the system for more than four weeks, and measured their actual use behaviour using the system usage log record.

Of the eight hypotheses, two were rejected (H2 and H5), four were accepted (H1, H3, H4, and H6), and one was partially accepted (H8a) as a result of the moderating effect tests for age and gender. Performance expectancy had significant positive effects on behavioural intention to use MyData. This result is consistent with those of previous studies on mHealth and eHealth services [33, 55, 56]. If users believe that they can derive healthcare management benefits from HiMD, their willingness to use it will be stronger. Therefore, to increase the behavioural intention of users on the HiMD platform, it is necessary for various partners and companies to provide services using personal health records to collaborate to provide functions that benefit users' healthcare.

Previous studies have shown that effort expectancy on behavioural intention to use has a significant effect on and is an important factor in behavioural intention to use [30, 31]. Contrariwise, the results of this study did not show any significant effect of effort expectancy on behavioural intention to use HiMD. This finding was unexpected; in other words, effort expectancy did not significantly affect a users’ intention to use the MyData platform, We might speculate on the reason for this. Effort expectancy might have no direct effect on usage behaviour; it may also have an indirect effect on user adoption through performance expectancy [55, 57]. However, we did not consider whether effort expectancy had an indirect effect in this study. Effort expectancy is a valuable factor in technology acceptance; therefore, developers should consider designing usable and easy-to-use user interfaces (UI). In addition, users considered systems with a well-designed UI that aligned with their needs as easy to understand and use [58]. Considering that user interface fit (UIF) positively affects effort expectancy, investigations into UIFs could also be conducted in a future study.

Social influence and facilitating conditions showed different results for each study. In this study, social influence and facilitating conditions had significant positive effects on behavioural intention to use HiMD, similar to the results of Park et al. [30] and Zhang et al. [55]. To reorganise this, facilitating conditions affected behavioural intention to use the HiMD, but it did not directly impact actual use behaviour. The influence of significant others on eHealth acceptance is an aspect of interactions among colleagues, other patients, and experts in clinical settings [59]. In other words, considering that social influence exerted an effect on behavioural intention to use HiMD, the MyData platform should expand functions that enable interaction between colleagues or healthcare experts. We have provided a variety of support to users, before and after using the platform, especially when they felt discomfort or experienced trouble. This method reduces the dropout rate of the users. System developers and planners should provide continuous assistance services and consider guidelines to support users. This is because facilitating conditions affect behavioural intention to use HiMD, and intention to use affects actual use behaviour. Many studies of healthcare systems using the UTAUT model have been conducted [30, 31], although they were limited to 'intention to use'. The contribution of this study is that we objectively measured 'actual use behaviour' using system log data. Scores were calculated according to the degree of use of the platform functions, such as consent, data check, data download, and data sharing. By combining subjective and objective data, we confirmed that intention to use significantly affects actual use behaviour.

The impact of the main factors in UTAUT was not moderated by age or gender, except for performance expectancy. Gender was partially supported as a moderator of performance expectancy, indicating that the effect of the link between performance expectancy and behavioural intention to use was significant for the male group but not for the female group. Our sample included more women than men. This is one of the limitations of this study, and this imbalance may have affected the results. However, as we only included participants who had used the HiMD, this gender gap might ne fairly representative of the HiMD user population, asin real life, women are more likely to engage in eHealth activities [60]. This result related to gender needs to be interpreted with caution because finding the main barriers to adopting a data-sharing system is highly important. Future work needs to departmentalise the factors influencing the use of HiMD and build various hypotheses regarding the relationships between the factors to identify additional influential factors.

Another limitation is that this study adopted the main factors of UTAUT. Therefore further research is needed to investigate this more thoroughly by adding perceived security to the model [50]. Additionally, the available personal health records utilised by the users were limited to health check-up data, drug prescription data, and depression scale testing data. Further studies with more general data are necessary.

Unlike one study in which MyData acceptance was rejected in most hypotheses [5], we confirmed many hypotheses of the UTAUT model for the MyData platform based on personal health record data sharing. Our results suggest that performance expectations for healthcare, social influence, and facilitating conditions modulate the acceptance of eHealth based on a personal health record data sharing system. This may account for the differences before and after the system was used. In addition, this study confirmed the usage behaviour of the MyData platform by utilising the system’s actual usage log for each function. The effect of intention to use on actual use was also analysed.

### Implications

Our study contributes significantly to the field of healthcare information technology in two ways. First, we applied the UTAUT model to users who used the MyData platform to identify the influencing consumers’ eHealth technology acceptance. Second, we conducted a previous study that confirmed the intention to use the system and evaluated the actual use behaviour using the system log.

### Limitations

First, data items should be expanded so that more data from the HIS can be linked for implemention within the platform. In addition, it is necessary to expand the functions of the platform and healthcare services provided. The expansion of this data sharing and utilisation platform will contribute to the construction of a data ecosystem. The research model should then be expanded to increase its explanatory power in predicting behavioural intention regarding the MyData platform, as by including additional factors of the UTAUT2 model and perceived security.

Second, each factor was separated like the original UTAUT factors, but the item loading of SI construction was lower than the others and was observed to have an association with PE in the analysis of the correlations. The correlation coefficient between the factor items checked, but the correlation coefficient of each item between PE and SI was below 0.71. This seems to indicate the possibility that the SI is related to the PE; further research is thus needed on this correlation.Third, studies have shown that of the characteristics of participants, education might mediate some of the relationships between UTAUT items and technology acceptance [61, 62]. We considered education as a moderating factor, but 95.4% of the platform users received education above college graduation. Most participants who visited the health examination centre at university hospitals had an education level above college graduate, and for this reason, we excluded education from the moderating factors. We need to consider and research various moderating factors in the future to enhance the acceptance of healthcare technology.

### Conclusions

This study is the first to examine the factors influencing the use of the MyData platform based on the personal health record data sharing system in Korea. In addition, this study is significant in using the eHealth system and in conductingat survey during the COVID-19 pandemic. In conclusion, the results of our study provide pioneering empirical evidence for the UTAUT model in the MyData platform-based personal health record data sharing system. In other words, we provide managerial insights that may increase acceptance and usage of the MyData platform. Focusing on the significant constructs that we found, future research may create other variants of the MyData platform to more appropriately provide various healthcare services.

The paradigm of healthcare has changed, and COVID-19 has accelerated this trend. MyData, which reflects the right to self-determination, is an important component of this paradigm shift. Personal data portability can create value by actively utilising data in the platform economy, and its expansion requires policy management, technical support, and user participation [10, 35]. This research will serve as a significant foundation for accepting data portability and data sharing concepts and expanding the data economy and data ecosystem. Finally, we plan to conduct future research using an expanded research model with the expansion of data sharing platforms.

## Supplementary Information

**Additional file 1**.** Supplementary Table 1**. Definition of the factors.** Supplementary Table 2**. Questionnaires.** Supplementary Table 3**. Log category for the system usage.** Supplementary Table 4**. Exploratory factor analysis.** Supplementary Table 5**. Reliability and convergent validity.** Supplementary Table 6**. Discriminant validity.** Supplementary Figure 1**. Parallel analysis scree plots.** Supplementary Figure 2**. Correlation matrix plot.

## Data Availability

The datasets generated and/or analysed during the current study are not publicly available because they contain system usage history, but are available from the corresponding author upon reasonable request.
