# Supplementary material for: Study of the factors influencing the use of MyData platform based on personal health record data sharing system
Source: BMC Med Inform Decis Mak. 2022 Jul 15;22:182. doi: 10.1186/s12911-022-01929-z (PMC9283557; doi:10.1186/s12911-022-01929-z)

BMC Medical Informatics and Decision Making

**Supplementary Information**

**Study on the factors influencing the use of MyData platform based on personal health record data sharing system**

Wona Choi^1^, Se-Hyun Chang^1^, Yoon-Sik Yang^1^, Surin Jung^1^, Seo-Joon Lee^1^, Ji-Won Chun^1^, Dai-Jin Kim^2^, Woojeong Lee^3^, In Young Choi^1*^

^1^Department of Medical Informatics, The Catholic University of Korea College of Medicine, Seoul, Republic of Korea

^2^Department of Psychiatry, Seoul St. Mary’s Hospital, The Catholic University of Korea College of Medicine, Seoul, Republic of Korea

^3^Department of Emergency Medicine, Incheon St. Mary’s Hospital, The Catholic University of Korea College of Medicine, Incheon, Republic of Korea

^*^Corresponding Author

In Young Choi

Department of Medical Informatics, The Catholic University of Korea College of Medicine, Seoul, Republic of Korea

[Postal address] 222 Banpo-daero, Seocho-gu, Seoul, Republic of Korea (06591)

[Phone number] 82-2-2258-7870

[Email address] iychoi@catholic.ac.kr

Supplementary Table 1. Definition of the factors

Supplementary Table 2. Questionnaires

Supplementary Table 3. Log category for the system usage

Supplementary Table 4. Exploratory factor analysis

Supplementary Table 5. Reliability and convergent validity

Supplementary Table 6. Discriminant validity

Supplementary Figure 1. Parallel analysis scree plots

Supplementary Figure 2. Correlation matrix plot

Supplementary Table 1. Definition of the factors

| Variable | Definition | No. of questions |
| --- | --- | --- |
| Total | | 27 |
| Performance Expectancy | The degree to which users believe that using the HiMD will help them improve their health | 4 |
| Effort Expectancy | The degree of ease associated with the use of the HiMD | 4 |
| Social Influence | The degree to which users feel that important relatives or work colleagues believe that the HiMD should be used for enhanced health management | 4 |
| Facilitating Conditions | The degree to which users believe that an organizational and technical infrastructure exists to support use of the HiMD | 4 |
| Behavioral intention to use | The degree of users’ behavioral intention to use the HiMD | 5 |
| General characteristics | Age, gender, education, income, experience | 6 |

Supplementary Table 2. Questionnaires

| Variable | Items | |
| --- | --- | --- |
| Performance Expectancy  (PE) | PE1 | Using ‘HiMD’ helps me improve my health care |
|  | PE2 | I know that 'HiMD' is useful for my health care |
|  | PE3 | 'HiMD' raises the level of my health care |
|  | PE4 | Using ‘HiMD’ helps improve health management performance |
| Effort Expectancy  (EE) | EE1 | It is easy to learn how to use ‘HiMD’ |
|  | EE2 | ‘HiMD’ can be used easily |
|  | EE3 | It is easy for me to learn the function and operation method of ‘HiMD’ |
|  | EE4 | It is easy for me to understand how to use ‘HiMD’. |
| Social Influence  (SI) | SI1 | People who influence me will encourage me to use HiMD |
|  | SI2 | People around me will think I should use ‘HiMD’ |
|  | SI3 | I think other people should use HiMD. |
|  | SI4 | I would be proud to use ‘HiMD’ |
| Facilitating Conditions  (FC) | FC1 | I can receive detailed guidance and manuals for using ‘HiMD’ |
|  | FC2 | I can receive related education in using ‘HiMD’ |
|  | FC3 | When difficulties arise in using ‘HiMD’, you can receive help and management from a professional person (group) |
|  | FC4 | Through ‘HiMD’, I can obtain effective knowledge and various experiences. |
| behavioral intention to use  (BI) | BI1 | I am willing to use ‘HiMD’ for healthcare in the future |
|  | BI2 | I will continue to use ‘HiMD’ for health management. |
|  | BI3 | I will try to use ‘HiMD’ frequently for healthcare |
|  | BI4 | I will try to use ‘HiMD’ more than other management systems for healthcare |
|  | BI5 | I am willing to recommend the use of ‘HiMD’ to those around me for healthcare. |

Supplementary Table 3. Log category for the system usage

| **Log** | **Detail** |
| --- | --- |
| Consent | Consent to share data, transfer data, use consent regarding third parties |
| Data check | Searching user’s data among selected hospital |
| Data download | Downloading data searched by users |
| Data sharing | Sharing data selected by users |

Supplementary Table 4. Exploratory factor analysis

| **Variable** | **Item** | **ML1** | **ML2** | **ML3** | **ML4** | **ML5** |
| --- | --- | --- | --- | --- | --- | --- |
| Performance Expectancy  (PE) | PE1 | 0.323 | 0.188 | 0.725 | 0.190 | 0.274 |
|  | PE2 | 0.329 | 0.226 | 0.757 | 0.203 | 0.215 |
|  | PE3 | 0.309 | 0.197 | 0.767 | 0.247 | 0.250 |
|  | PE4 | 0.345 | 0.221 | 0.767 | 0.196 | 0.242 |
| Effort Expectancy  (EE) | EE1 | 0.158 | 0.792 | 0.205 | 0.280 | 0.130 |
|  | EE2 | 0.204 | 0.805 | 0.211 | 0.245 | 0.143 |
|  | EE3 | 0.167 | 0.883 | 0.128 | 0.214 | 0.117 |
|  | EE4 | 0.187 | 0.883 | 0.160 | 0.238 | 0.146 |
| Social Influence  (SI) | SI1 | 0.382 | 0.248 | 0.426 | 0.257 | 0.597 |
|  | SI2 | 0.356 | 0.188 | 0.347 | 0.256 | 0.689 |
|  | SI3 | 0.411 | 0.223 | 0.415 | 0.262 | 0.617 |
|  | SI4 | 0.399 | 0.200 | 0.364 | 0.278 | 0.614 |
| Facilitating Conditions  (FC) | FC1 | 0.232 | 0.335 | 0.211 | 0.725 | 0.178 |
|  | FC2 | 0.260 | 0.318 | 0.175 | 0.778 | 0.217 |
|  | FC3 | 0.265 | 0.287 | 0.212 | 0.738 | 0.174 |
|  | FC4 | 0.335 | 0.327 | 0.265 | 0.657 | 0.196 |
| Behavioral Intention  (BI) | BI1 | 0.753 | 0.222 | 0.330 | 0.258 | 0.197 |
|  | BI2 | 0.794 | 0.211 | 0.311 | 0.263 | 0.246 |
|  | BI3 | 0.797 | 0.211 | 0.309 | 0.250 | 0.242 |
|  | BI4 | 0.734 | 0.202 | 0.315 | 0.258 | 0.263 |
|  | BI5 | 0.707 | 0.217 | 0.335 | 0.281 | 0.310 |

Supplementary Figure 1. Parallel analysis scree plot


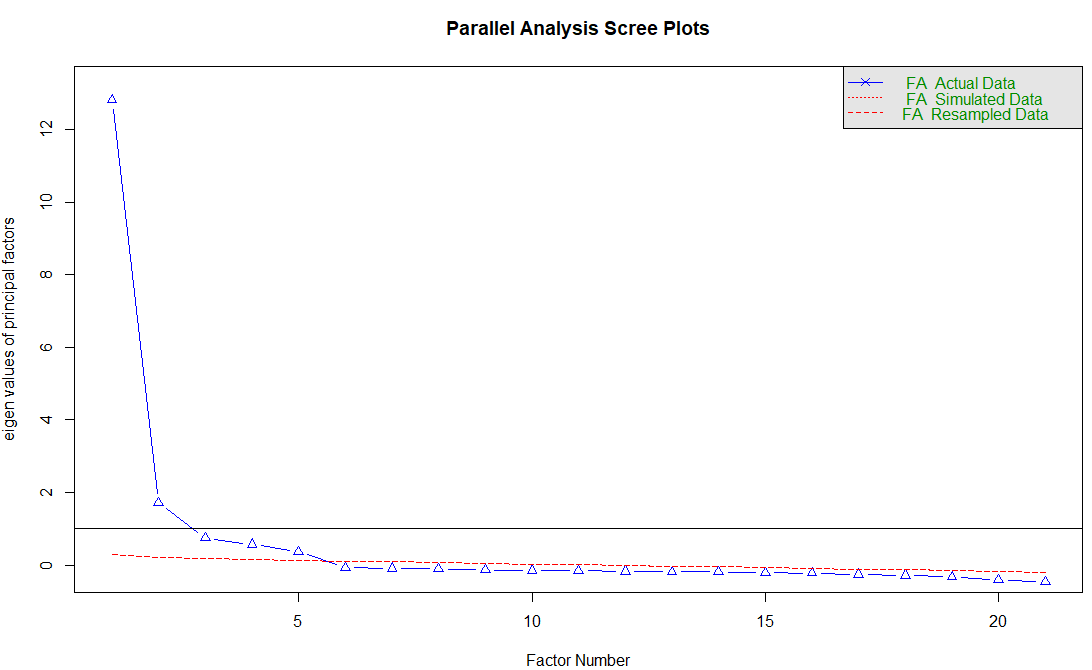


**Supplementary Table 5. Reliability and convergent validity**

| **Variable** | **Item** | **Factor loading** | **Cronbach’s α (>0.7)** | **CR (>0.7)** | **AVE (>0.5)** |
| --- | --- | --- | --- | --- | --- |
| Performance Expectancy  (PE) | PE1 | 0.881 | 0.949 | 0.949 | 0.823 |
|  | PE2 | 0.903 |  |  |  |
|  | PE3 | 0.918 |  |  |  |
|  | PE4 | 0.924 |  |  |  |
| Effort Expectancy  (EE) | EE1 | 0.901 | 0.944 | 0.945 | 0.808 |
|  | EE2 | 0.890 |  |  |  |
|  | EE3 | 0.924 |  |  |  |
|  | EE4 | 0.885 |  |  |  |
| Social Influence  (SI) | SI1 | 0.894 | 0.953 | 0.953 | 0.835 |
|  | SI2 | 0.909 |  |  |  |
|  | SI3 | 0.928 |  |  |  |
|  | SI4 | 0.925 |  |  |  |
| Facilitating Conditions  (FI) | FC1 | 0.870 | 0.935 | 0.935 | 0.783 |
|  | FC2 | 0.912 |  |  |  |
|  | FC3 | 0.879 |  |  |  |
|  | FC4 | 0.876 |  |  |  |
| Behavioural Intention  (BI) | BI1 | 0.908 | 0.966 | 0.967 | 0.853 |
|  | BI2 | 0.947 |  |  |  |
|  | BI3 | 0.943 |  |  |  |
|  | BI4 | 0.905 |  |  |  |
|  | BI5 | 0.913 |  |  |  |
| Model measurement fits: χ2=897.244 (df=179, p=<0.001), CFI=0.976, TLI=0.971, RMSEA=0.059, SRMR=0.024 | | | | | |
| CR: composite reliability, AVE: average variance extracted | | | | | |

**Supplementary Table 6. Discriminant validity**

|  | **PE** | **SI** | **EE** | **FC** | **BI** |
| --- | --- | --- | --- | --- | --- |
| **PE** | 0.907 |  |  |  |  |
| **SI** | 0.826 | 0.899 |  |  |  |
| **EE** | 0.536 | 0.564 | 0.914 |  |  |
| **FC** | 0.64 | 0.714 | 0.679 | 0.885 |  |
| **BI** | 0.772 | 0.829 | 0.548 | 0.707 | 0.924 |
| PE: Performance Expectancy, EE: Effort Expectancy, SI: Social Influence,  FC: Facilitating Conditions, BI: Behavioural Intention, AUB: Actual Use Behaviour | | | | | |

Supplementary Figure 2. Correlation matrix plot


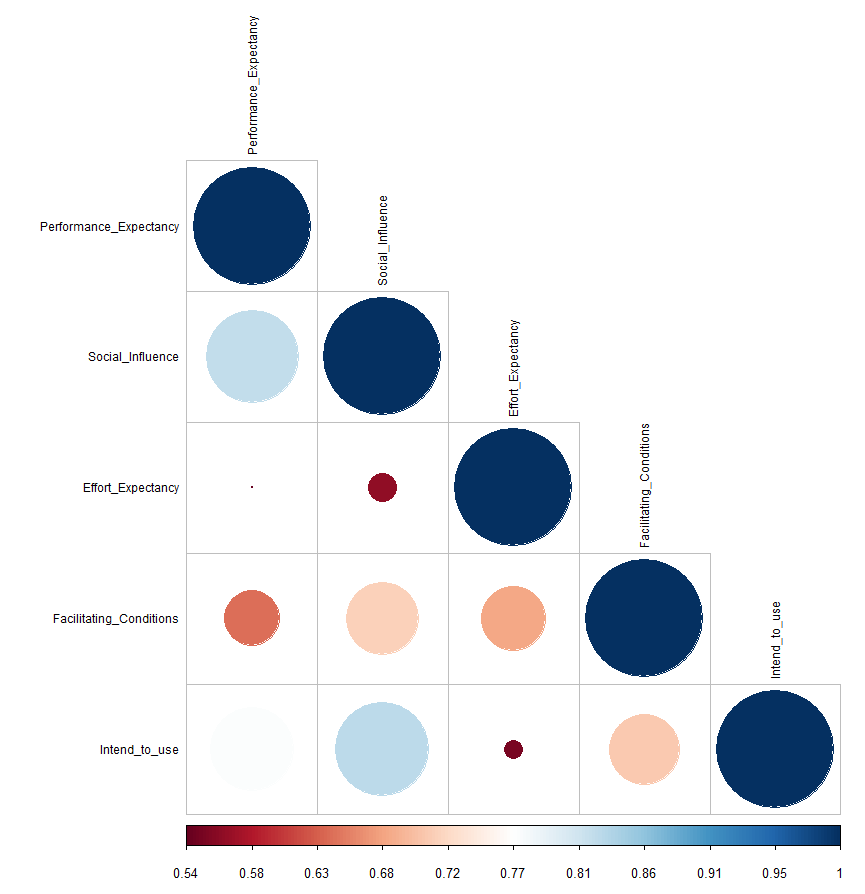

Supplement: Supplementary file 1 — Additional file 1. Supplementary Table 1. Definition of the factors. Supplementary Table 2. Questionnaires. Supplementary Table 3. Log category for the system usage. Supplementary Table 4. Exploratory factor analysis. Supplementary Table 5. Reliability and convergent validity. Supplementary Table 6. Discriminant validity. Supplementary Figure 1. Parallel analysis scree plots. Supplementary Figure 2. Correlation matrix plot. [file 12911_2022_1929_MOESM1_ESM.docx]
